# Genetic Variation in PFKFB3 Impairs Antifungal Immunometabolic Responses and Predisposes to Invasive Pulmonary Aspergillosis

**DOI:** 10.1128/mBio.00369-21

**Published:** 2021-05-28

**Authors:** Samuel M. Gonçalves, Daniela Antunes, Luis Leite, Toine Mercier, Rob ter Horst, Joana Vieira, Eduardo Espada, Carlos Pinho Vaz, Rosa Branca, Fernando Campilho, Fátima Freitas, Dário Ligeiro, António Marques, Frank L. van de Veerdonk, Leo A. B. Joosten, Katrien Lagrou, Johan Maertens, Mihai G. Netea, João F. Lacerda, António Campos, Cristina Cunha, Agostinho Carvalho

**Affiliations:** a Life and Health Sciences Research Institute (ICVS), School of Medicine, University of Minho, Braga, Portugal; b ICVS/3B’s - PT Government Associate Laboratory, Guimarães/Braga, Portugal; c Serviço de Transplantação de Medula Óssea (STMO), Instituto Português de Oncologia do Porto, Porto, Portugal; d Department of Hematology, University Hospitals Leuven, Leuven, Belgium; e Department of Microbiology, Immunology and Transplantation, KU Leuven, Leuven, Belgium; f Department of Internal Medicine, Radboud University Nijmegen Medical Centre, Nijmegen, the Netherlands; g Radboud Center for Infectious Diseases (RCI), Radboud University Nijmegen Medical Centre, Nijmegen, the Netherlands; h Serviço de Hematologia e Transplantação de Medula, Hospital de Santa Maria, Lisbon, Portugal; i Instituto Português do Sangue e Transplantação (IP), Porto, Portugal; j Instituto Português do Sangue e Transplantação (IP), Lisbon, Portugal; k Serviço de Imuno-Hemoterapia, Hospital de Braga, Braga, Portugal; l Clinical Department of Laboratory Medicine, University Hospitals Leuven, Leuven, Belgium; m National Reference Center for Mycosis, University Hospitals Leuven, Leuven, Belgium; n Department for Genomics & Immunoregulation, Life and Medical Sciences Institute (LIMES), University of Bonn, Bonn, Germany; o Instituto de Medicina Molecular, Faculdade de Medicina da Universidade de Lisboa, Lisbon, Portugal; Universidade de Sao Paulo

**Keywords:** immunometabolism, PFKFB3, *Aspergillus*, invasive pulmonary aspergillosis, stem cell transplantation, single nucleotide polymorphism, macrophage, antifungal immunity

## Abstract

Activation of immune cells in response to fungal infection involves the reprogramming of their cellular metabolism to support antimicrobial effector functions. Although metabolic pathways such as glycolysis are known to represent critical regulatory nodes in antifungal immunity, it remains undetermined whether these are differentially regulated at the interindividual level. In this study, we identify a key role for 6-phosphofructo-2-kinase/fructose-2,6-bisphosphatase 3 (PFKFB3) in the immunometabolic responses to Aspergillus fumigatus. A genetic association study performed in 439 recipients of allogeneic hematopoietic stem cell transplantation (HSCT) and corresponding donors revealed that the donor, but not recipient, rs646564 variant in the *PFKFB3* gene increased the risk of invasive pulmonary aspergillosis (IPA) after transplantation. The risk genotype impaired the expression of PFKFB3 by human macrophages in response to fungal infection, which was correlated with a defective activation of glycolysis and the ensuing antifungal effector functions. In patients with IPA, the risk genotype was associated with lower concentrations of cytokines in the bronchoalveolar lavage fluid samples. Collectively, these findings demonstrate the important contribution of genetic variation in *PFKFB3* to the risk of IPA in patients undergoing HSCT and support its inclusion in prognostic tools to predict the risk of fungal infection in this clinical setting.

## INTRODUCTION

Recent advances in medicine and the introduction of broad-spectrum antibiotics have prompted an increasing incidence of invasive fungal infections, particularly among hematological patients with chemotherapy or patients undergoing solid organ or allogeneic hematopoietic stem cell transplantation (HSCT) (1). Invasive pulmonary aspergillosis (IPA) is a major cause of mortality in these clinical settings, with rates estimated between 20 and 30% (2, 3) but that can reach 80% when infection involves azole-resistant strains (4). The clinical relevance of these infections is further emphasized by limitations in available tests for the diagnosis of IPA. The development of more effective medical interventions is therefore an urgent need that demands an improved understanding of the pathogenesis of IPA.

The reprogramming of immune cell metabolism is acknowledged as a crucial event required for mounting protective immune responses against fungal pathogens (5). During infection, innate immune cells, e.g., monocytes and macrophages, rewire their energy metabolism from oxidative phosphorylation to glycolysis to rapidly and efficiently support antimicrobial functions, such as phagocytosis and production of inflammatory mediators (6). The critical role of glucose metabolism during infection is further supported by experimental evidence demonstrating that its blockade dampens the immune response and hampers the clearance of selected bacterial and fungal pathogens (7–11). In turn, pathogens have also developed complex virulence strategies to exploit and subvert the metabolic requirements of immune cells to their benefit (12, 13).

The recognition of pathogen-associated molecular patterns (PAMPs) drives substantial changes in cellular metabolism and effector functions of immune cells (14). The fungal cell wall contains a considerable diversity of PAMPs, including β-1,3-glucan, melanin, and chitin (15, 16), capable of promoting the metabolic and functional reprogramming of immune cells. For example, stimulation of monocytes with β-1,3-glucan promotes metabolic and epigenetic changes underlying the acquisition of a “trained immunity” phenotype characterized by enhanced cytokine production in response to heterologous secondary stimulation (7, 17–20). More recently, the removal of fungal melanin within the phagosomal compartment of macrophages was established as the activating signal required for the induction of glucose metabolism in immune cells during infection with Aspergillus fumigatus (8).

The upregulation of glycolytic enzymes upon infection also directly supports cytokine expression through mechanisms that involve “moonlighting” activities (21, 22). The 6-phosphofructo-2-kinase/fructose-2,6-bisphosphatase 3 (PFKFB3) enzyme is a master regulator of the glycolytic pathway (23). By orchestrating the balance between the synthesis and degradation of fructose-2,6-bisphosphate, it acts as an allosteric activator of 6-phophofructo-1-kinase, the rate-limiting enzyme in glycolysis (24). As a result of its glycolysis-promoting activity, PFKFB3 has been shown to coordinate antiviral effector functions of macrophages (25) and to be highly expressed in myeloid cells from critically ill coronavirus disease 2019 (COVID-19) patients (26). Also, PFKFB3 has been shown to be a target of the zinc fingers and homeoboxes 2 (ZHX2) transcription factor and, as a result, to accelerate disease progression in models of sepsis (27).

Since the risk of infection varies considerably even among patients with comparable immune dysfunction and predisposing clinical factors, susceptibility to IPA is thought to depend largely on genetic predisposition (28–32). However, the potential involvement of variation in genes involved in glucose metabolism during the development of IPA has never been addressed. Here, we describe the association of genetic variation in *PFKFB3* with an increased risk for developing IPA in HSCT patients as the result of a defective metabolic reprogramming and downstream antifungal effector functions of macrophages. Collectively, our results pinpoint a novel genetic factor regulating metabolic responses in immune cells and provide support for risk stratification and preventive measures aimed at a more effective management of IPA.

## RESULTS

### The *PFKFB3* locus influences cytokine production during fungal infection.

We have recently demonstrated that activation of glycolysis is required for effective immune responses against A. fumigatus (8). We, therefore, examined whether variation in genes involved in the glycolytic pathway affected host responses to fungal infection. To do so, we tested the association of common single nucleotide polymorphisms (SNPs) (minor allele frequency, ≥5%) in relevant candidate genes with differential cytokine production after fungal stimulation of peripheral blood mononuclear cells (PBMCs) from healthy individuals of the 500FG cohort in the Human Functional Genomics Project (HFGP).

**FIG 1:**
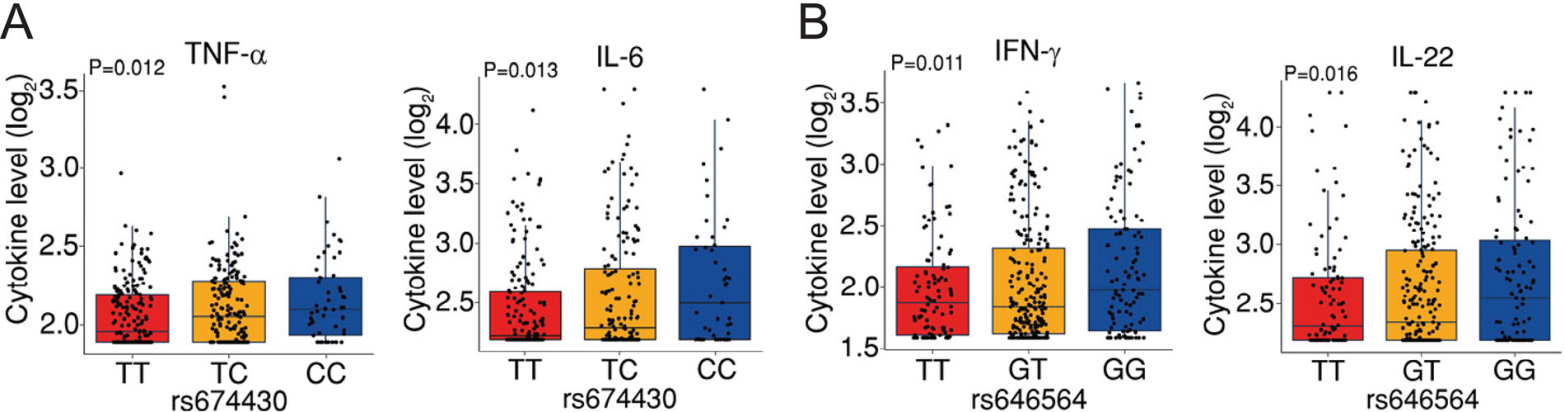
The *PFKFB3* locus influences the production of cytokines by PBMCs. (A) Levels (log_2_) of TNF and IL-6 according to rs674430 genotypes, and (B) IFN-γ and IL-22 according to rs646564 genotypes detected after stimulation of PBMCs from the 500FG cohort with A. fumigatus for 24 h or 7 days, respectively. Data are expressed as mean values ± standard errors of the means (SEM). Overall *P* values were determined using a linear regression model with age and gender as covariates.

We identified a total of 107 cytokine quantitative trait loci (cQTLs) in three genes involved in glucose metabolism, namely, *PFKFB3*, *HK2*, and *AKT1* (see Table S1 in the supplemental material). Although these were not different at a genome-wide level, several strong cQTLs were identified in *PFKFB3* displaying an uncorrected *P* value of <0.05. We next assessed linkage disequilibrium (LD) between the SNPs in *PFKFB3* (see Fig. S1 in the supplemental material) and identified rs674430 and rs646564 to represent suitable candidates tagging the largest LD blocks influencing the production of multiple cytokines (Table S1). Specifically, the rs674430 SNP was associated with a genotype-dependent effect on the production of tumor necrosis factor (TNF) (overall *P* = 0.012) and interleukin 6 (IL-6) (overall *P* = 0.013) by PBMCs after 24 h of infection (Fig. 1A), whereas the rs646564 SNP influenced gamma interferon (IFN-γ) (overall *P* = 0.011) and IL-22 (overall *P* = 0.016) produced by cells stimulated for 7 days (Fig. 1B). Collectively, these findings suggest genetic variation in *PFKFB3* as an important regulator of cytokine production in response to A. fumigatus infection.

10.1128/mBio.00369-21.1 FIG S1 Linkage disequilibrium structure of the *PFKFB3* gene. The localizations of the *PFKFB3* SNPs were obtained from the human genome assembly GRCh37 (hg19) and are indicated with vertical lines. The dbSNP reference numbers are indicated for each SNP. Blue colors refer to *r*^2^, and red colors refer to D’. The intensity of the coloration reflects the degree of LD. Download FIG S1, EPS file, 2.9 MB. Copyright © 2021 Gonçalves et al. 2021 Gonçalves et al. https://creativecommons.org/licenses/by/4.0/ This content is distributed under the terms of the Creative Commons Attribution 4.0 International license.

10.1128/mBio.00369-21.2 TABLE S1 Analysis of cytokine QTLs in PBMCs stimulated with A. fumigatus. Download Table S1, XLSX file, 0.01 MB. Copyright © 2021 Gonçalves et al. 2021 Gonçalves et al. https://creativecommons.org/licenses/by/4.0/ This content is distributed under the terms of the Creative Commons Attribution 4.0 International license.

### Genetic variation at the *PFKFB3* locus increases the risk of IPA after HSCT.

**FIG 2:**
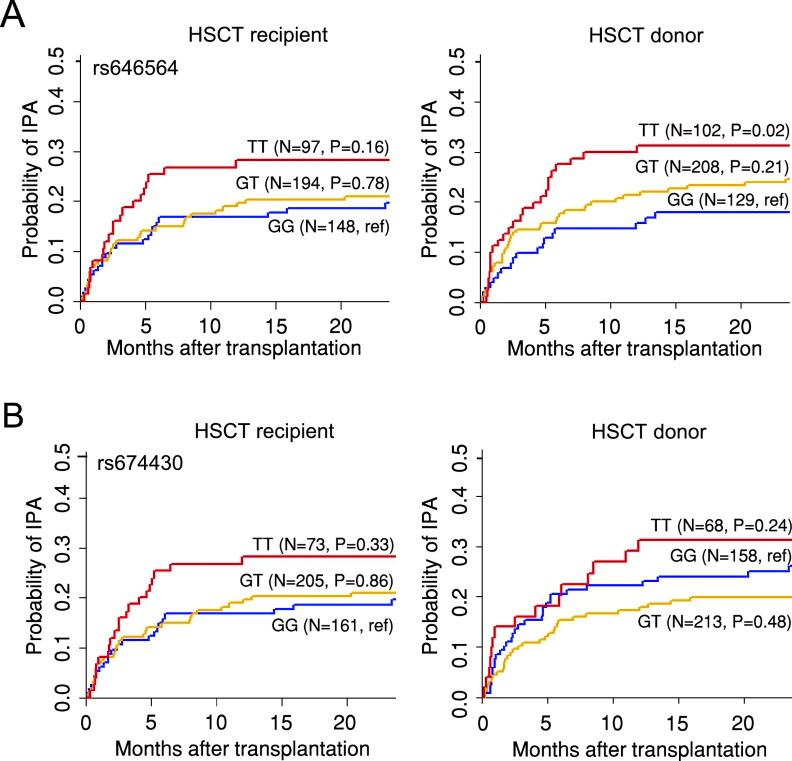
Genetic variation in *PFKFB3* influences the risk of IPA. Cumulative incidence of IPA in 439 eligible HSCT recipients from the IFIGEN cohort according to the recipient or donor *PFKFB3* genotypes at rs646564 (A) and rs674430 (B). Data were censored at 24 months, and relapse and death were competing events. *P* values are for Gray’s test.

Because of the critical role of SNPs in *PFKFB3* in regulating cytokine production, we next investigated the relationship between genetic variants at this locus and susceptibility to IPA in a disease-relevant context. We, therefore, assessed the cumulative incidence of IPA in patients from the IFIGEN cohort undergoing allogeneic HSCT (Table 1) according to recipient or donor *PFKFB3* genotypes at the rs674430 or rs646564 SNPs. Our results demonstrate that the donor, but not the recipient, rs646564 was associated with an increased risk of IPA after transplantation (Fig. 2A). The cumulative incidence of IPA for donor rs646564 was 31% for TT (*P* = 0.02), 23% for GT (*P* = 0.21), and 18% for GG genotypes (reference), respectively. In contrast, no significant association with the risk of IPA was detected for rs6744330 (Fig. 2B). In a multivariate model accounting for patient age and gender, hematological malignancy, type of transplantation, conditioning regimen, development of acute graft-versus-host-disease (GVHD) grade III-IV and antifungal prophylaxis, the donor TT genotype conferred a 2.7-fold increased risk of developing IPA (*P* = 0.0017) (Table 2). These results highlight genetic variation at the *PFKFB3* locus in the donor compartment as a critical risk factor regulating susceptibility to IPA after HSCT.

**TABLE 1:** Baseline characteristics of transplant recipients enrolled in the study^*a*^

Variable^*b*^	Value for transplant recipients^*c*^		*P* value
	With IPA (*n* = 91)	Without IPA (*n* = 348)	
Age at transplantation, no. (%)			
≤20 yrs	13 (14.3)	69 (19.8)	0.264
21–40 yrs	23 (25.3)	101 (29.0)	
>40 yrs	55 (60.4)	178 (51.2)	
Gender, no. (%)			
Female	38 (41.8)	150 (43.1)	0.859
Male	53 (58.2)	198 (56.9)	
Underlying disease, no. (%)			
Acute leukemia	49 (53.8)	179 (51.5)	0.115
Lymphoproliferative diseases	14 (15.4)	69 (19.8)	
Myelodysplastic/myeloproliferative diseases	13 (14.3)	30 (8.6)	
Chronic myeloproliferative diseases	7 (7.7)	20 (5.7)	
Aplastic anemia	6 (6.6)	17 (4.9)	
Other	2 (2.2)	33 (9.5)	
Transplantation type, no. (%)			
Matched, related	34 (37.4)	169 (48.6)	0.037
Matched, unrelated	33 (36.3)	81 (23.3)	
Mismatched, related	0 (0.0)	7 (2.0)	
Mismatched, unrelated	24 (26.4)	91 (26.2)	
Graft source, no. (%)			
Peripheral blood	80 (87.9)	287 (82.5)	0.506
Bone marrow	10 (11.0)	53 (15.2)	
Cord blood	1 (1.1)	8 (2.3)	
Disease stage, no. (%)			
First complete remission	49 (53.8)	188 (54.0)	0.800
Second or subsequent remission, or relapse	13 (14.3)	59 (17.0)	
Active disease	29 (31.9)	101 (29.0)	
Conditioning regimen, no. (%)			
RIC	68 (74.7)	228 (65.5)	0.091
Myeloablative	23 (25.3)	120 (34.5)	
CMV serostatus of donor and recipient, no. (%)			
D−/R+ or D+/R+	80 (87.9)	313 (89.9)	0.504
D−/R− or D+/R−	11 (12.1)	35 (10.1)	
Duration of neutropenia,^*d*^ mean days (range)	13.1 (8–39)	13.5 (5–35)	0.460
Acute GVHD, no. (%)			
No GVHD or grades I–II	63 (69.2)	302 (86.8)	0.0002
Grades III–IV	28 (30.8)	46 (13.2)	
Antifungal prophylaxis, no. (%)^*e*^			
Fluconazole	42 (46.2)	117 (33.6)	0.002
Posaconazole	26 (28.6)	107 (30.8)	
Other	9 (9.9)	14 (4.0)	
None or unknown	14 (15.4)	110 (31.6)	

a Twenty-one patients with “possible” IPA were excluded. Lymphoproliferative diseases included cases of chronic lymphocytic leukemia, multiple myeloma, and B- and T-cell lymphomas. Chronic myeloproliferative diseases included cases of chronic myelogenous leukemia and primary myelofibrosis. Other diseases included cases of idiopathic medullar aplasia, lymphohistiocytosis, hemoglobinopathies, and paroxysmal nocturnal hemoglobinuria.

b RIC, reduced intensity conditioning; CMV, cytomegalovirus; D, donor; R, recipient; GVHD, graft-versus-host-disease.

c The number of transplant recipients (percentage) with the characteristic are shown.

d Neutropenia was defined as ≤0.5 × 10^9^ cells/liter.

e Other antifungals used in prophylaxis included voriconazole, liposomal amphotericin B, itraconazole and caspofungin. *P* values were calculated by Fisher’s exact probability *t* test or Student’s *t* test for continuous variables.

**TABLE 2:** Multivariate analysis of the association between rs646564 in *PFKFB3* and risk of IPA^*a*^

Genetic or clinical variable	Adjusted HR^*b*^ (95% CI) (*n* = 439)	*P* value
Donor TT at rs646564	2.72 (1.46–5.06)	0.0017
aGVHD III-IV	3.84 (1.46–10.1)	0.0062

a Multivariate analyses were based on the subdistribution regression model of Fine and Gray. aGVHD, acute graft-versus-host-disease.

b Hazard ratios were adjusted for patient age and gender, hematological malignancy, type of transplantation, conditioning regimen, development of acute GVHD (aGVHD) grade III-IV and antifungal prophylaxis. Only the variables remaining significant after adjustment are shown. HR, hazard ratio; CI, confidence interval.

### The rs646564 SNP impairs PFKFB3-mediated activation of glycolysis.

**FIG 3:**
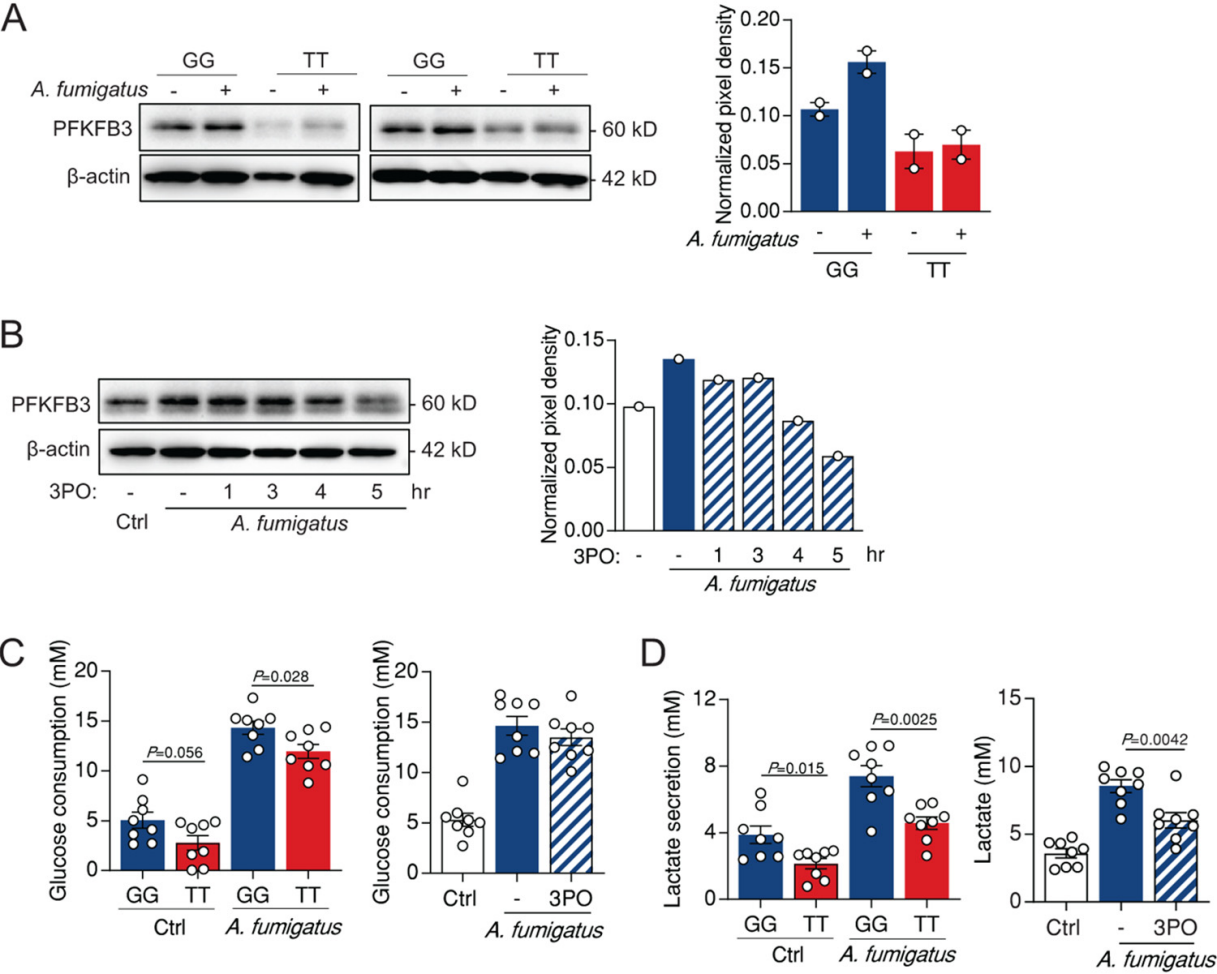
The rs646564 SNP in *PFKFB3* inhibits the activation of glycolysis in macrophages. (A and B) Expression of PFKFB3 in macrophages infected with A. fumigatus for 2 h according to different rs646564 genotypes (A) or following treatment with 3PO (representative of three independent experiments) (B). The pixel density of the PFKFB3 was normalized to β-actin. (C and D) Glucose consumption (C) and lactate secretion (D) by macrophages left untreated or infected with A. fumigatus for 24 h, according to different rs646564 genotypes or following treatment with 3PO. Data are expressed as mean values ± SEM. Ctrl, control.

To understand the mechanisms through which rs646564 might impact the metabolic homeostasis of immune cells and contribute to antifungal immune responses, we next assessed the expression of PFKFB3 in macrophages from healthy donors carrying different rs646564 genotypes after infection with A. fumigatus. In line with its enhanced transcriptional activity (8), the expression of the PFKFB3 protein was also increased early after infection (Fig. 3A). However, induction of PFKFB3 was instead markedly impaired in macrophages carrying the TT genotype compared to cells from GG carriers. The treatment with 3-(3-pyridinyl)-1-(4-pyridinyl)-2-propen-1-one (3PO), despite its role as a selective inhibitor of PFKFB3, also promoted a slight decrease in the expression of PFKFB3 (Fig. 3B). In line with its impact on PFKFB3 expression, the rs646564 SNP was found to significantly impair glucose consumption by both unstimulated and 24-h-infected macrophages with the TT genotype (Fig. 3C). Macrophages from TT carriers also secreted smaller amounts of lactate than cells with the GG genotype under the same conditions (Fig. 3D), a finding ultimately pointing to a defective activation of the glycolytic pathway in carriers of the T allele. In support of these findings, inhibition of PFKFB3 with 3PO resulted in effects similar to those of the TT genotype on lactate secretion (Fig. 3D), although not glucose consumption (Fig. 3C). These results demonstrate that the rs646564 SNP contributes to IPA by compromising the efficient activation of glycolysis by macrophages in response to fungal infection.

### The rs646564 SNP compromises antifungal effector functions of macrophages.

**FIG 4:**
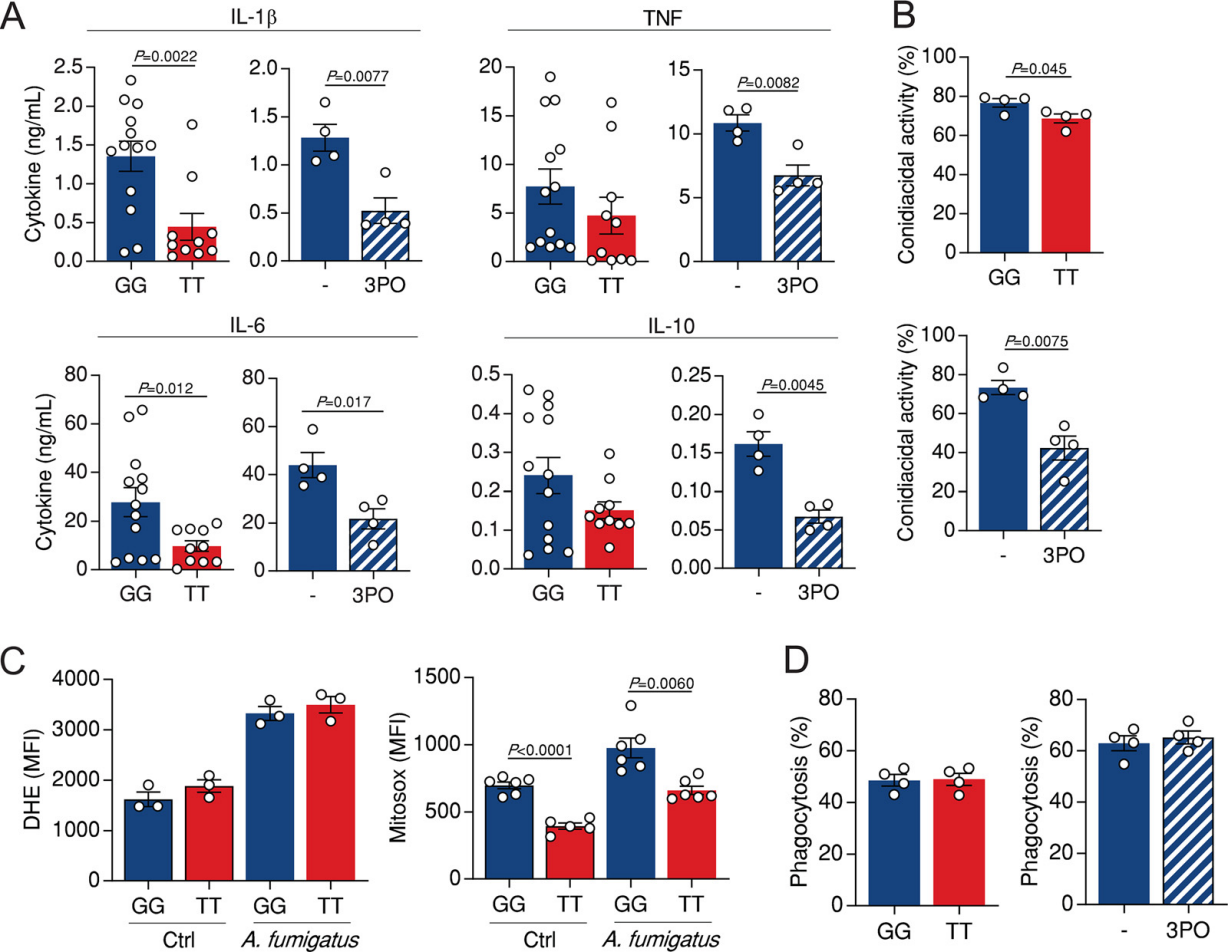
Antifungal effector mechanisms of macrophages are impaired by the rs646564 SNP in *PFKFB3*. (A) Production of IL-1β, TNF, IL-6, and IL-10 by macrophages infected with A. fumigatus for 24 h according to different rs646564 genotypes or following treatment with 3PO. (B) Conidiacidal activity of macrophages according to different rs646564 genotypes or following treatment with 3PO. (C) Production of cytosolic ROS (left) and mitochondrial ROS (right) by macrophages infected with A. fumigatus for 4 h according to different rs646564 genotypes or following treatment with 3PO. Data are expressed as mean fluorescence intensity (MFI). DHE, dihydroethidium. (D) Phagocytosis of macrophages according to different rs646564 genotypes or following treatment with 3PO. Data are expressed as mean values ± SEM.

To mount a protective immune response during infection with A. fumigatus, effector functions of macrophages such as the production of proinflammatory cytokines, phagocytosis, and killing activity are required (16). We tested therefore how the impairment of glycolysis driven by the rs646564 SNP in *PFKFB3* might affect these antifungal mechanisms. Besides influencing the production of the adaptive cytokines IFN-γ and IL-22 (Fig. 1B), the TT genotype also impaired the secretion of proinflammatory cytokines, but not IL-10, by macrophages after 24 h of infection (Fig. 4A), a finding illustrating likely distinct regulatory mechanisms mediated by rs646564 across cell types. A comparable defect in cytokine secretion by infected macrophages was also observed after the pharmacological inhibition of PFKFB3 (Fig. 4A). In addition, the conidiacidal activity of macrophages from TT carriers was also significantly decreased compared to cells with the GG genotype (Fig. 4B), an effect that could, at least in part, be explained by the impaired production of reactive oxygen species (ROS) by TT macrophages after infection with A. fumigatus (Fig. 4C). Likewise, the conidiacidal activity (Fig. 4B) was also similarly impaired upon treatment with 3PO. In contrast, the phagocytic ability of macrophages was not compromised in either the presence of the TT genotype or 3PO-treated macrophages (Fig. 4D). These results confirm a critical role for PFKFB3 in the induction and regulation of antifungal effector functions of macrophages in response to A. fumigatus.

### Genetic variation at the *PFKFB3* locus regulates the alveolar cytokine profile in IPA.

**FIG 5:**
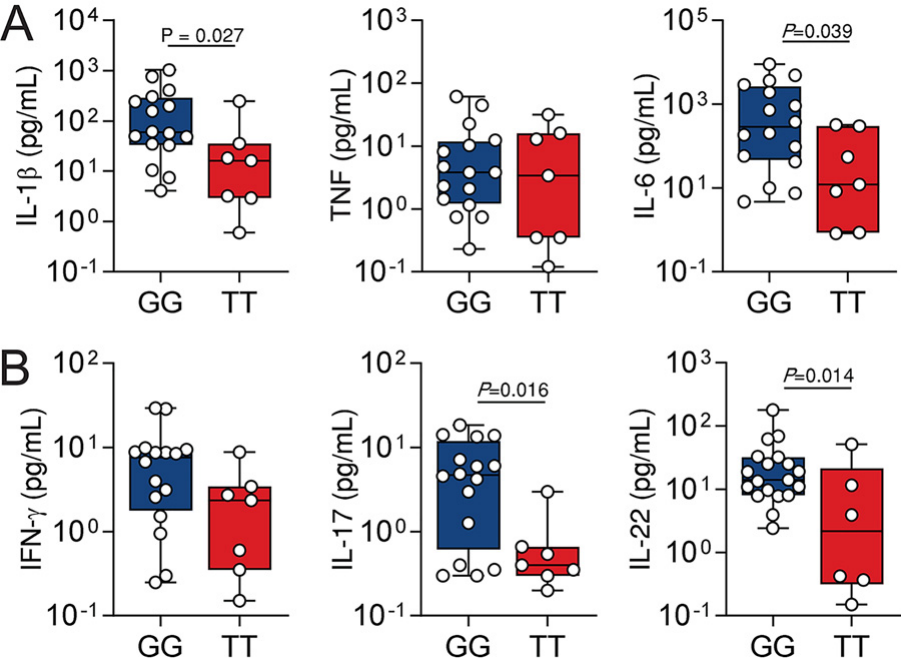
PFKFB3 regulates cytokine production in IPA. (A and B) Levels of IL-1β, TNF, and IL-6 (A) and IFN-γ, IL-17, and IL-22 (B) in BAL fluid samples from patients diagnosed with IPA (*n* = 16 for GG; *n* = 7 for TT). Data are expressed as mean values ± SEM.

To examine whether the rs646564 SNP also regulated cytokine production in IPA patients, we next profiled the concentration of relevant cytokines in bronchoalveolar lavage fluid (BAL) samples collected from patients enrolled in the FUNBIOMICS study at diagnosis of fungal infection (33). We found that the alveolar concentrations of IL-1β and IL-6 were lower among patients carrying the TT genotype than GG carriers (Fig. 5A). In line with their regulation by the rs646564 SNP in PBMCs (Fig. 1B), production of T cell-derived cytokines was also influenced, with lower concentrations of IL-17A and IL-22 detected in samples from TT carriers compared to GG carriers (Fig. 5B). Collectively, these findings illustrate a critical link between genetic variation in the glycolytic pathway and the activation of immune responses in patients with IPA and may explain the association with susceptibility to infection.

## DISCUSSION

Several factors are known to predispose to fungal disease, particularly in immunocompromised and severely ill patients, but these alone fail to explain the development of infection in all affected patients. Several studies have reported an expanding number of common SNPs associated with the development of IPA in at-risk patients (28, 30). On the basis of their validation across independent cohorts (32), the most robust markers for IPA identified thus far include well-known components of the immune response to A. fumigatus, such as the soluble pattern recognition molecule pentraxin-3 (PTX3) (29, 32) and the C-type lectin receptor dectin-1 (31, 34). However, no evidence disclosing a relevant contribution of the host genetic profile to the immunometabolic responses activated in response to A. fumigatus has been disclosed thus far. We now demonstrate that genetic variation in *PFKFB3* contributes to IPA via molecular mechanisms influencing the metabolic homeostasis of immune cells and the signals that orchestrate cytokine production and fungal clearance.

To counter fungal infection, myeloid cells undergo metabolic reprogramming via distinct mechanisms whereby enhanced glycolysis fuels antimicrobial effector functions required for effective clearance of the pathogen (8, 19). A key step in the regulation of the glycolytic pathway is the PFKFB3-mediated control of the levels of the fructose-2,6-bisphosphate metabolite. Our data disclose a genetic variant associated with defective activation of PFKFB3 following fungal infection. These results are in line with previous reports demonstrating that the genetic inactivation of PFKFB3 in murine cells was shown to disrupt glycolysis and innate defenses against other infections such as infection by the respiratory syncytial virus (25). Likewise, the defective induction of PFKFB3 in T cells from rheumatoid arthritis patients was reported to drive a hypoglycolytic phenotype, impairing ATP generation, the redox balance, and the production of ROS. In contrast, overexpression of PFKFB3 in patient T cells enhances the glycolytic flux and protects cells from excessive apoptosis (35). These findings highlight PFKFB3 as a critical regulatory node at the interface of metabolism and immunity.

We demonstrate that the activation of PFKFB3 is critical for the regulation of cytokine production and antifungal effector functions in macrophages. The activation of the NLRP3 inflammasome in macrophages and the subsequent release of IL-1β have been found to modulate glycolysis via PFKFB3 (36). Importantly, genetic variation in PFKFB3 is correlated with differential production of IL-1β after fungal infection, suggesting the existence of bidirectional signaling events, in which PFKFB3 may also be able to regulate the activation of the NLRP3 inflammasome to drive the production of IL-1β. Endothelial PFKFB3 expression was also found to be increased after TNF treatment (37), and IL-6 was reported to enhance glycolysis in mouse embryonic fibroblasts and human cell lines by upregulating PFKFB3 expression (38). These results suggest the existence of a reciprocal regulation between production of proinflammatory cytokines and PFKFB3 activation. However, where the cross talk between these mechanisms occurs remains unclear. Recent reports highlight that, although the activity of PFKFB3 as a major regulator of glycolysis occurs mostly in the cytosol, it is also able to mediate glycolysis-independent nuclear roles (27). This suggests that PFKFB3 can, directly or indirectly, modulate and interact with the NF-κB pathway to regulate inflammatory responses.

PFKFB3 has a largely detrimental role in supporting cancer cell growth, by promoting glycolysis, cell cycle progression, and angiogenesis (39). In this sense, and although the inactivation of PFKFB3 is regarded as a promising therapeutic opportunity across several types of cancer (24, 40), we hypothesize that the balanced induction of this enzyme specifically in immune cells may instead represent a valuable strategy benefiting patients suffering from IPA, especially those harboring loss-of-function SNPs in *PFKFB3*. Accordingly, the activation of PFKFB3 has been shown to accelerate disease progression in models of sepsis (27). In this context, the inhibition of PFKFB3 could be therapeutically exploited to dampen the pathogenic activation of glycolysis, and therefore, it is not surprising that such approaches have been shown to alleviate sepsis-related acute lung injury by suppressing inflammatory responses and the apoptosis of alveolar epithelial cells (41).

Current limitations in the management of fungal diseases, as well as concerns over the emergence of antifungal resistance, are inspiring the search for novel host-directed therapies. Our expanded understanding of how metabolic networks coordinate immune cell function and how specific genetic signatures influence immunometabolic responses and regulate susceptibility to fungal disease is opening new horizons toward personalized medical interventions based on individual genomics. The renewed interest in immunometabolism is expected to improve our understanding of how these signaling networks coordinate immune cell function, ultimately paving the way toward innovative therapeutic approaches involving adjuvant immunotherapy to reorient host cells toward immune protection in the context of fungal infections.

## MATERIALS AND METHODS

### Ethics statement.

Approval for the IFIGEN study was obtained from the Ethics Subcommittee for Life and Health Sciences (SECVS) (no. 125/014), the Ethics Committee for Health of the Instituto Português de Oncologia, Porto, Portugal (no. 26/015), the Ethics Committee of the Lisbon Academic Medical Center, Portugal (no. 632/014), and the National Commission for the Protection of Data, Portugal (no. 1950/015). The FUNBIOMICS study was approved by SECVS (no. 126/2014) and the Ethics Committee of the University Hospitals of Leuven, Belgium. The Human Functional Genomics Project (HFGP) study was approved by the Ethical Committee of Radboud University Nijmegen, the Netherlands (no. 42561.091.12). Experiments were conducted according to the principles expressed in the Declaration of Helsinki, and participants provided written informed consent. The functional experiments involving cells isolated from the peripheral blood of healthy volunteers at the Hospital of Braga, Portugal, was approved by the SECVS of the University of Minho, Portugal (no. 014/015).

### Population cohorts.

The functional experiments were performed in healthy individuals of Western European ancestry recruited at the Hospital de Braga, Braga, Portugal, and in the 500FG cohort from the HFGP, which comprises 534 Dutch healthy individuals of Western European ancestry. The genetic association study with IPA was performed with a total of 460 hematological patients of European ancestry undergoing allogeneic HSCT at Instituto Português de Oncologia, Porto, Portugal, and at Hospital de Santa Maria, Lisbon, Portugal, enrolled in the IFIGEN study between 2009 and 2015. Of these, 439 had available donor and recipient DNA samples and patient-level data. The demographic and clinical characteristics of the patients are summarized in Table 1. Ninety-one cases of probable/proven IPA were identified according to the standard criteria from the European Organization for Research and Treatment of Cancer/Mycology Study Group (EORTC/MSG) (42). Twenty-one patients were excluded from the study based on the “possible” classification of infection.

### SNP selection and genotyping.

Genomic DNA was isolated from whole blood using the QIAcube automated system (Qiagen). SNPs were selected based on their putative role as cytokine QTLs in the HFGP study and on their ability to tag surrounding variants with a pairwise correlation coefficient *r*^2^ of at least 0.80 and a minor allele frequency of ≥5% using publicly available sequencing data from Pilot 1 of the 1000 Genomes Project for the CEU population. Genotyping of rs674430 and rs646564 SNPs in *PFKFB3* was performed using KASPar assays (LGC Genomics) in an Applied Biosystems 7500 fast real-time PCR system (Thermo Fisher Scientific), according to the manufacturer’s instructions. The DNA samples of individuals from the 500FG cohort were genotyped using the commercially available chip Illumina HumanOmniExpressExome-8 v1.0. Quality control and imputation were performed as described previously (43).

### Cytokine QTL mapping.

Following the generation of genotype and cytokine data, cytokine QTLs were mapped as described previously (43). Briefly, concentrations of human cytokines were determined using specific commercial enzyme-linked immunosorbent assay (ELISA) kits (PeliKine Compact, or R&D Systems), per the manufacturer’s instructions. Cytokine QTLs were identified by log-transforming raw cytokine levels and mapping them to genotype data using a linear regression model with age, gender, and cell counts as covariates.

### Generation of monocyte-derived macrophages.

Buffy coats from healthy donors were obtained after written informed consent. Briefly, peripheral blood mononuclear cells (PBMCs) were enriched from buffy coats by density gradient using Histopaque-1077 (Sigma-Aldrich). Cells present in the enriched mononuclear fraction were washed twice in phosphate-buffered saline (PBS) and resuspended in RPMI 1640 culture medium with 2 mM glutamine (Thermo Fisher Scientific) supplemented with 10% human serum (Sigma-Aldrich), 10 U/ml penicillin-streptomycin, and 10 mM HEPES (Thermo Fisher Scientific). Monocytes were then separated using positive magnetic bead separation with anti-CD14^+^-coated beads (MACS Miltenyi) according to the manufacturer’s instructions. To evaluate the purity of the isolated monocyte population, 5 × 10^5^ cells were stained with a BV 650 anti-human CD14 (clone M5E2) antibody for 30 min at 4°C. Cell viability was assessed by staining with Zombie Green fluorescent dye (BioLegend) for 30 min at 4°C. Pellets were washed and resuspended in fluorescence-activated cell sorting (FACS) buffer (PBS containing 2% fetal bovine serum [FBS] and 2 mM EDTA) prior to analysis. Data were obtained on a BD FACS LSRII instrument (Becton Dickinson) and processed using FlowJo (Tree Star Inc.). The obtained CD14^+^ populations displayed a purity higher than 94% and yielded more than 97% viable cells. Isolated monocytes were then resuspended in complete RPMI medium and seeded at a concentration of 1 × 10^6^ cells/ml in 24-well and 96-well plates (Corning Inc.) for 7 days in the presence of 20 ng/ml of recombinant human granulocyte-macrophage colony-stimulating factor (GM-CSF; Miltenyi Biotec). Acquisition of macrophage morphology was confirmed by visualization with a BX61 microscope (Olympus, Tokyo, Japan).

### Stimulation of MDMs.

Unless otherwise indicated, monocyte-derived macrophages (MDMs) (5 × 10^5^/well in 24-well plates) were either left untreated or stimulated with live conidia of A. fumigatus at a 1:5 or 1:10 effector-to-target ratio, respectively, for 24 h at 37°C and 5% CO_2_. In some conditions, MDMs were pretreated for 4 h with 30 μM 3-(3-pyridinyl)-1-(4-pyridinyl)-2-propen-1-one (3PO) to inhibit PFKFB3 activity. In all experiments, data were assessed in triplicates and are shown as the mean value for each individual.

### Quantification of glucose and lactate by HPLC.

After infection, supernatants were removed, centrifuged, and transferred to high-performance liquid chromatography (HPLC) tubes. Glucose and lactate levels were determined using a Gilson pump system (Gilson) with a 54°C HyperREZ XP Carbohydrate H^+^ 8-μm (Thermo Fisher Scientific) column and a refractive index detector (IOTA 2; Reagents). The mobile phase consisting of 0.0025 M H_2_SO_4_ was filtered and degasified for at least 45 min before use. Standard solutions were prepared in MilliQ water (Millipore). All data were analyzed using the Gilson Uniprot software, version 5.11.

### Phagocytosis assay.

To evaluate phagocytosis, MDMs (5 × 10^5^/well, in 24-well plates) were infected with fluorescein isothiocyanate (FITC)-labeled conidia of A. fumigatus at a 1:5 effector-to-target ratio. The infection was synchronized for 30 min at 4°C, and phagocytosis was initiated by shifting the coincubation to 37°C at 5% CO_2_ for 1 h. Phagocytosis was stopped by washing wells with PBS, and extracellular conidia were stained with 0.25 mg/ml Calcofluor White (Sigma-Aldrich) for 15 min at 4°C to avoid further ingestion. After the wells were washed twice with PBS, the cells were fixed with PBS containing 3.7% (vol/vol) formaldehyde for 15 min. The number of MDMs with ingested green conidia was enumerated by examining the slides by fluorescence microscopy (Olympus), and data were expressed as the percentage of MDMs that internalized one or more conidia.

### Conidiacidal activity assay.

Following differentiation, MDMs (1 × 10^5^/well, in 96-well plates) were stimulated with live A. fumigatus conidia at a 10:1 effector-to-target ratio for 1 h at 37°C and 5% CO_2_ to allow the internalization of conidia. Medium containing the noningested conidia was removed, and wells were washed twice with PBS. To measure the conidiacidal activity, MDMs were allowed to eliminate the internalized conidia for 2 h at 37°C in 5% CO_2_. After incubation, culture plates were snap-frozen at −80°C and thawed at 37°C to cause cell lysis and release of ingested conidia. Serial dilutions of cell lysates were plated on solid growth media, and following a 24-h incubation at 37°C, the number of colony-forming units (CFU) was enumerated, and the percentage of CFU inhibition was calculated.

### Measurement of ROS production.

MDMs (5 × 10^5^/well, in 24-well plates) were infected with live A. fumigatus conidia at a 1:5 effector-to-target ratio for 4 h at 37°C and 5% CO_2_. After infection, the culture medium was removed, and cells were trypsinized for 10 min at 37°C. Then, cells were collected and centrifuged for 5 min at 2,000 rpm. Finally, each pellet was resuspended in 10 μM dihydroethidium (cytosolic ROS) or 5 μM Mitosox (mitochondrial ROS) (Thermo Fisher Scientific), and cells were then incubated for 30 min at 37°C, protected from light. Data were obtained on a BD FACS LSRII instrument (Becton Dickinson) and processed using FlowJo (Tree Star Inc.).

### ELISA.

MDMs (5 × 10^5^/well in 24-well plates) were infected with A. fumigatus conidia at a 1:5 or 1:10 effector-to-target ratio, respectively, for 24 h at 37°C and 5% CO_2_. After infection, supernatants were collected, and cytokine levels were quantified using ELISA MAX Deluxe Set kits (BioLegend), according to the manufacturer’s instructions. Cytokine measurements in BAL samples were performed using the Human Premixed Multi-Analyte kit (R&D Systems), according to the manufacturer’s instructions.

### Western blot analysis.

MDMs (5 × 10^5^/well, in 24-well plates) were infected with A. fumigatus conidia for 2 h at a 1:10 effector-to-target ratio at 37°C in 5% CO_2_. After infection, cells were lysed in radioimmunoprecipitation assay (RIPA) buffer (50 mM Tris, 250 mM NaCl, 2 mM EDTA, 1% NP-40, 10% glycerol [pH 7.2], and a mixture of protease inhibitors [Roche Molecular Biochemicals]). Cell lysis was performed at 4°C for 30 min (with shaking), and samples were then centrifuged. The protein content was determined using the Bradford dye-binding (Bio-Rad) method. Laemmli buffer (Bio-Rad) was added to 20 μg of protein, and samples were boiled and separated on a 12% sodium dodecyl sulfate-polyacrylamide gel (SDS-PAG) and transferred to nitrocellulose membranes (Bio-Rad). Western blotting was performed according to the manufacturer’s instructions, using the following primary antibodies: rabbit anti-PFKFB3 and mouse anti-β-actin, both from Abcam and diluted 1:1,000. Secondary antibodies used were anti-rabbit (Thermo Fisher Scientific) and anti-mouse (Bio-Rad), both diluted to 1:5,000. The blots were developed using chemiluminescence (SuperSignal West Femto maximum sensitivity substrate; Thermo Fisher Scientific) and detected with ChemiDoc XRS system (Bio-Rad). Signal intensities and quantifications were determined with the ImageLab 4.1 analysis software (Bio-Rad).

### BAL fluid collection.

BAL specimens were collected as previously described (33). Briefly, specimens were collected using a flexible fiber-optic bronchoscope following local anesthesia with 2% lidocaine (Xylocaine), when infection was clinically suspected. Samples were obtained by instillation of a prewarmed 0.9% sterile saline solution (20 ml twice). The sampling area was determined based on the localization of the lesion on chest imaging (X-ray or computed tomography scan). BAL specimens with comparable recovery rates were used. All samples were stored at −80°C until use.

### Statistical analysis.

The probability of IPA according to *PFKFB3* genotypes was determined using the cumulative incidence method and compared using Gray’s test (44). Cumulative incidences at 24 months were computed with the *cmprsk* package for R version 2.10.1 (45), with censoring of data at the date of the last follow-up visit and relapse and death as competing events. All clinical and genetic variables achieving a *P* value of ≤0.15 in the univariate analysis were entered one by one in a pairwise model together and kept in the final model if they remained significant (*P* < 0.05). Multivariate analysis was performed using the subdistribution regression model of Fine and Gray with the *cmprsk* package for R (46). Data obtained in functional assays were expressed as means ± standard errors of the means (SEM). Unpaired Student’s *t* test with Bonferroni’s adjustment and two-tailed Mann-Whitney rank sum test were used to determine statistical significance (*P* < 0.05).
